# Bluetongue Viruses Based on Modified-Live Vaccine Serotype 6 with Exchanged Outer Shell Proteins Confer Full Protection in Sheep against Virulent BTV8

**DOI:** 10.1371/journal.pone.0044619

**Published:** 2012-09-25

**Authors:** René G. P. van Gennip, Sandra G. P. van de Water, Mieke Maris-Veldhuis, Piet A. van Rijn

**Affiliations:** Central Veterinary Institute of Wageningen UR (CVI), Department of Virology, AB Lelystad, The Netherlands; Federal University of Pelotas, Brazil

## Abstract

Since 1998, Bluetongue virus (BTV)-serotypes 1, 2, 4, 9, and 16 have invaded European countries around the Mediterranean Basin. In 2006, a huge BT outbreak started after incursion of BTV serotype 8 (BTV8) in North-Western Europe. IN 2008, BTV6 and BTV11 were reported in the Netherlands and Germany, and in Belgium, respectively. In addition, Toggenburg orbivirus (TOV) was detected in 2008 in Swiss goats, which was recognized as a new serotype of BTV (BTV25). The (re-)emergency of BTV serotypes needs a rapid response to supply effective vaccines. Reverse genetics has been developed for BTV1 and more recently also for BTV6. This latter strain, BTV6/net08, is closely related to live-attenuated vaccine for serotype 6 as determined by full genome sequencing. Here, we used this strain as backbone and exchanged segment 2 and 6, respectively Seg-2 (VP2) and Seg-6 (VP5), for those of BTV serotype 1 and 8 using reverse genetics. These so-called ‘serotyped’ vaccine viruses, as mono-serotype and multi-serotype vaccine, were compared for their protective capacity in sheep. In general, all vaccinated animals developed a neutralizing antibody response against their respective serotype. After challenge at three weeks post vaccination with cell-passaged, virulent BTV8/net07 (BTV8/net07/e1/bhkp3) the vaccinated animals showed nearly no clinical reaction. Even more, challenge virus could not be detected, and seroconversion or boostering after challenge was negligible. These data demonstrate that all sheep were protected from a challenge with BTV8/net07, since sheep of the control group showed viremia, seroconversion and clinical signs that are specific for Bluetongue. The high level of cross-protection is discussed.

## Introduction

Bluetongue virus (BTV) belongs to the family *Reoviridae*, genus *Orbivirus* [1]. BTV transmission between ruminants, including cattle, sheep, and goats, occurs in majority by bites of species of *Culicoides*. Bluetongue (BT) is listed as a ‘notifiable disease’ by the Office International des Epizooties (OIE) [2] causing severe hemorrhagic disease with fever, lameness, coronitis, swelling of the head (particularly the lips and tongue) and death. Twenty-four BTV serotypes have been recognized as defined by cross-neutralization assays, and recent BTV isolates are considered as serotype 25 and 26, which was partially based on sequence data [3], [4].

The genome of BTV consists of ten linear double-stranded RNA genome segments Seg-1 to Seg-10 encoding structural proteins VP1 to VP7, nonstructural proteins, NS1, NS2 and NS3/NS3a, for reviews see [5], [6], and the recently discovered nonstructural protein NS4 [7], [8]. The virus particle composes three shells of proteins, the inner shell consists of VP3 encoded by Seg-3, the middle shell consists of VP7 encoded by Seg-7, and the outer shell is formed by VP2 (Seg-2) and VP5 (Seg-6). The BTV particle further contains three enzymatic proteins, VP1 (Seg-1), VP4 (Seg-4) and VP6 (Seg-9), and one copy of each of the ten genome segments Seg-1 to Seg-10 in the inner shell. The nonstructural proteins NS1 (Seg-5), NS2 (Seg-8), NS3/NS3a (Seg-10), and NS4 (Seg-9) are not part of the BTV particle.

Since 1998, BTV serotypes 1, 2, 4, 9, and 16 have invaded European countries around the Mediterranean Basin. In 2006, a huge BT outbreak started after incursion of BTV serotype 8 (IAH collection nr. BTV-8 NET2006/04) [9] in N-W Europe [10]. More recently, BTV serotypes 6 and 11 were reported in N-W Europe in 2008 [11], [12], [13], [14]. Both BTV strains are closely related to modified-live vaccine strains for their respective serotypes [13], [15]. In the same time period, an unknown orbivirus named Toggenburg orbivirus (TOV or BTV25) was discovered in Swiss goats [3].

Because of the repeated incursion of different serotypes there is a need for vaccines which can be applied as soon as these are available. The vaccination campaign for BTV serotype 8 (BTV8) in the northern part of Europe was launched in 2008, two years after the outbreak has started and after the devastating year 2007. Further, by the incursion or discovery of new serotypes, like BTV serotypes 25 and 26, it is evident that a rapid response to supply vaccines of desired serotypes is needed.

One way to address this problem, is by use of the recently developed reverse genetics system as described for BTV1 [16], BTV6 [17], and BTV8 [7], [17]. The reverse genetics system was used to genetically modify BTV [7], [17], [18], [19] and to generate reassortants [20].

Here, we used reverse genetics for vaccine-related BTV6/net08 [14] to exchange the outer shell for that of serotype 1 or 8 resulting in so-called ‘serotyped’ vaccine viruses. These vaccines, used as mono- or as multi-serotype vaccine, are completely protective for a challenge with virulent BTV8.

## Materials and Methods

### Cell lines and virus

BSR cells (a clone of BHK-21 cells [21]) were cultured in Dulbecco's modified Eagle's medium (DMEM; Invitrogen) containing 5% fetal bovine serum (FBS), 100 IU/ml penicillin, 100 μg/ml streptomycin and 2,5 ug/ml Amphotericin B.

All virus stocks were obtained by infection of BSR cells at low multiplicity of infection (MOI) and harvested after 100% cytopathic effect (CPE) was observed. Virus titers were determined by endpoint dilution and expressed as plaque forming units per ml (pfu/ml). Virus stocks were stored at −80°C.

### Rescue of BTVs by transfection of plasmid-derived RNA transcripts to mammalian cells

Transfection experiments with T7-derived RNA transcripts from linearized plasmids were performed as previously described for rgBTV1, 6 and 8 [17]. Briefly, monolayers of 10^5^ BSR cells per 2 cm^2^ were transfected with 600 ng equimolar amounts of RNA of BTV segments encoding VP1, VP3, VP4, NS1, VP6, NS2. In total, 600 ng RNA was transfected using 1 µg lipofectamine^TM^ 2000 (1∶2.5; 1 mg/ml Invitrogen) in Opti-MEM® I Reduced Serum Medium according to manufacturer's conditions. Eighteen to twenty hours post transfection, monolayers were transfected again with 600 ng equimolar amounts of ten BTV RNA segments. For serotyped BTV6 with the outer shell of BTV1 or 8, T7-derived RNA transcripts of Seg-2 and Seg-6 from serotype 1 or 8 were added to complete the set of ten RNA transcripts. At 4 hrs post transfection, the transfection mix was replaced with 1 ml DMEM supplemented with 5% FBS. Supernatants were harvested from monolayers developing cytopathogenic effect (CPE) at 48 hrs after the second transfection. BTV-specific CPE was confirmed by immunostaining of fixed monolayers with monoclonal antibody (Mab) produced by ATCC-CRL-1875 directed against VP7 according to standard procedures. Serotyped vaccine viruses with the outer shell of BTV1 or BTV8 were named BTVac-1 and BTVac-8, respectively. According to the names of these serotyped vaccine viruses (BTVac-x, x indicates the originating serotype of the outer shell proteins), previously rescued rgBTV6 [17] was here renamed BTVac-6.

### Growth curve of BTVac-6, BTVac-1 and BTVac-8 on BSR cells

Confluent BSR-monolayers in M24-well plates were infected in duplicate at a multiplicity of infection (MOI) of 0.1. After attachment to cells for 1.5 h at 37°C, the medium was removed and refreshed with 1 ml of DMEM with 5% FBS, 1% Penicillin/Streptomycin/Fungizone and incubation was continued. At 24, 48, 72 and 96 h post infection (hpi), samples of the supernatants were harvested and stored at −80°C. Virus titers were determined by endpoint dilution on BSR cells. Therefore, BSR cells were infected with tenfold dilutions of samples, and grown for 72 h. Positive wells were detected by immunostaining with VP7-specific Mab ATCC-CRL-1875. Virus titers were expressed as tissue culture infective dose (^10^logTCID)_50_/ml.

### Vaccination/challenge experiment with serotyped BTV viruses

All experiments with live animals were performed under the guidelines of the European Community (86/609) and were approved by the Committee on the Ethics of Animal Experiments of the Central Veterinary Institute (Permit Number: 2011-003). Twenty female Blessumer sheep of 6–24 months old and free of BTV and BTV-antibodies were commercially sourced from the same flock of a Dutch farm. The sheep were randomly allocated to five groups of four animals. Although it was intended to inject the animals with 1 ml virus with a titer of 10^5^ TCID_50_/ml, BTVac-6 appeared to have a much lower virus titer when the inoculum was re-titrated afterwards. Therefore, on day 0 (0 dpi), four sheep were intramuscularly (i.m.) vaccinated in the neck with either 1 ml of 10^1.4^ TCID_50_/ml BTVac-6, 1 ml of 10^5^ TCID_50_/ml BTVac-1 or BTVac-8. The fourth group (CombiVac group) was vaccinated i.m. with 1 ml in total consisting of 0.33.10^1.4^ TCID_50_/ml BTVac-6, 0.33.10^5^ TCID_50_/ml BTVac-1 and 0.33.10^5^ TCID_50_/ml BTVac-8. The fifth group served as control group. EDTA-blood samples were collected daily during the first week after inoculation and every other day until challenge at day 21. Serum samples were collected daily in the second week and every other day in the first and third week after immunization. Animals in all five groups were challenged with a total of 1 ml of 10^5^ TCID_50_/ml of BTV8/net07/e1/bhkp3 [17], here named BTV8/net07. Virus was injected subcutaneously (s.c.) to sheep between the shoulder blades left and right from the spinal cord. EDTA-blood samples were collected daily during the first week after challenge and every other day until the end of the experiment at 21 days post challenge. Serum samples were collected daily in the second week and every other day in the first and third week after challenge. EDTA-blood samples were tested by the in-house panBTV-PCRtest [22], [23]. Serum samples were tested by blocking ELISA (ID.VET) for the detection of BTV-specific antibodies in serum. Body temperature was recorded daily and fever was defined as the average temperature plus two times the standard deviation. Clinical signs were daily recorded according to the clinical score table for BTV8 animal trials (Table S1). Clinical signs were quantified following challenge by an adapted clinical reaction index (CRI) as described by Huismans [24]. A maximum score of 12 was given to the cumulative total of fever readings (a) as described above from days 3 to 14 post challenge (dpc), a clinical score (0–3) for each parameter according the clinical score table to a maximum score of 27 (b). An additional 4 points were added to the sum of a and b if death occurred within 14 dpi. The relative reaction (RR) is the CRI of the test sheep expressed as a percentage of that of the control. The percentage protection index  = 100– RR.

### Serum neutralisation test (SNT)

SNT was performed according to the method of Haig [25] using BTV1/bsrp3 (rgBTV1), BTV8/net07/e1/bhkp3 (BTV8/net07) and BTV6/Net08/e1/bhkp3/bsrp2 (BTV6/Net08). Briefly, sera were diluted (1∶10–1∶10,240) and titrated against 30–300 TCID_50_ of the abovementioned BTV strains for 1 h at 37°C. Then, 100 μl of a BSR cell suspension (2×10^5^/ml) was added per well and, after incubation for 6–7 days at 37°C, the wells were scored for cytopathic effect (CPE). The titer of neutralising antibodies (nAb titer) was determined as the dilution of serum giving a 50% neutralisation endpoint. Samples with nAb titers of >10 were considered positive.

### Seg-2 and Seg-6 specific PCR tests

Virus stocks and selected RNA samples of previously tested EDTA-blood samples by the panBTV-PCR test were analysed with in-house developed serotype specific real time PCR tests for serotypes 1, 6, and 8 (Table 1, [14]) targeting Seg-2 (S2-genotyping). Virus stocks of rescued BTVac-x vaccine viruses were also tested for the presence of Seg-6 RNA with PCR tests with primers that discriminate between Seg-6 of BTV1, −6 or −8, respectively (Table 1).

**Table 1:** Primers used for differential detection of genome segment Seg-2 (S2-genotyping) and Seg-6 of BTV serotype 1, 6, and 8.

name^a^	sequence	expected amplicon size (bp)
BTV1VP2/283-1F	TTGTTGAAAGTACGAGACACAAGAG	175
BTV1VP2/457-1R	GTATCAGCCTTCTTTGAATCGATT	
BTV1.1VP2 probe	CATCCACTGCACCCACTGGTCA	
		
BTV6VP2/1757-5F	AGGAACAGTCGGCTTATCAC	192
BTV6VP2/1948-5R	TTCGCTAATGTGCTTCTCCAT	
BTV6.5 VP2 probe	TTGTCAGCTTTACGCAAACCCCG	
		
BTV8VP2/1873-4F	CGGAGACAGCGCAGTATGTA	225
BTV8VP2/2097-4R	CCTCGGTAGTATCCCTCACG	
BTV8.4 VP2 probe	ACATACGATGCCYTCGGAGGATTCTG	
		
BTV1S6F1	AGTGATCAATGCTTTAAGCGGG	831
BTV1S6R	GTAAGTGTAAGTGCTTCCCGTC	
		
BTV6S6F1	GTTAAAAAGATCCCCATGAT	490
BTV6S6R4	CGCTTTACAGAGCACCTTAT	
		
BTV8S6F	GTTAAAAAAGCGATCGCTTTCG	638
BTV8S6R4	GCCTCTTTTAACGCATCG	

a. Primers were purchased with Eurogentec Benelux. Probes were purchased with TibMolBiol Berlin and are labeled with FAM at the 5′-end, and with the black hole quencher (BHQ) at the 3′-end.

### Detection of vaccine virus and challenge virus

**Figure 1:**
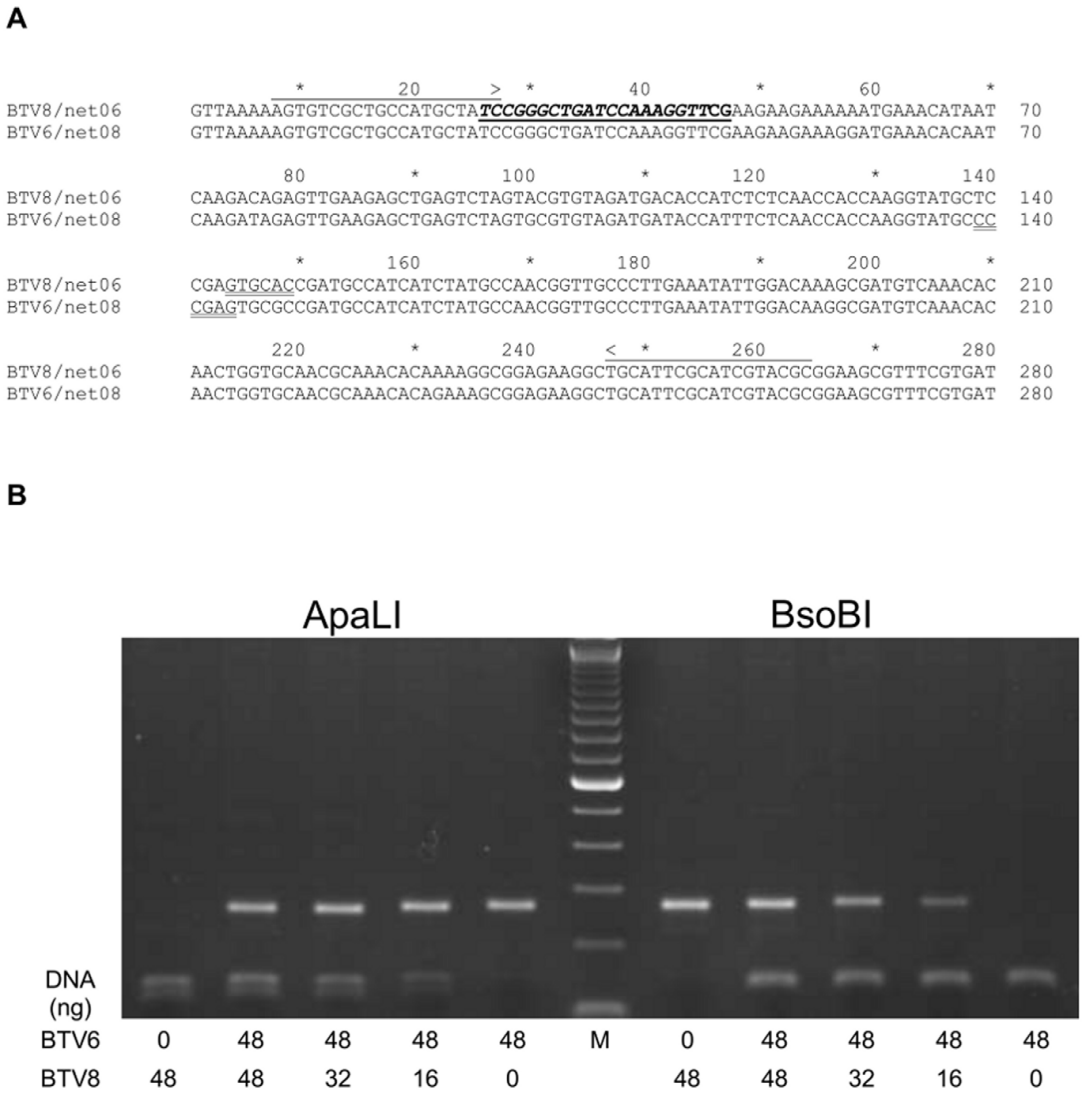
S10-genotyping by restriction analysis. (A) Sequence comparison of Seg-10 amplicons of BTVac-6 and BTV8/net07. Positions and orientations of primers and probe (bold, italics, underlined) are indicated by arrows [23]. The unique restriction sites of *Bso*BI (CYCGRG) en *Apa*LI (GTGCAC) are double underlined. (B) Mixing experiment of different amounts (ng) of Seg-10 amplicon from BTV6- or BTV8 digested with *Bso*BI or *Apa*LI and analysed by electrophoresis on a 1,5% agarose gel.

The Seg-10 amplicon as detected by the diagnostic panBTV-PCR test [23] of samples taken on 7, 14 and 21 dpc were further analysed to differentiate between Seg-10 RNA from BTV8 and from BTVac-x (S10-genotyping). Note that Seg-10 RNA from all BTVac vaccine viruses originates from BTV6/net08. From samples that were tested positive by the panBTV-PCR test, 5 µl amplicon was digested with *Bso*BI (specific for Seg-10 of BTVac-x) or with *Apa*LI (specific for Seg-10 of BTV8/net07), see Fig. 1. Digested amplicons were analysed on a 1.5% agarose gel to discriminate between amplicons derived from Seg-10 from BTVac-x vaccine viruses and from challenge virus BTV8/net07. The detection limit of digested amplicon was <16 ng (Fig. 1), whereas 5 ng of undigested amplicon can be detected (not shown).

## Results

### Reassortants with the outer shell of different serotypes

Since rgBTV6 (here named BTVac-6) was proven to be avirulent [14], we studied the possibilities of using this avirulent BTVac-6 as virus backbone to generate ‘synthetic’ reassortants with the outer shell originating from BTV1 or BTV8/net07. Previously, Seg-2 and Seg-6 sequences of BTV1 (genbank accession numbers FJ969721 and FJ969723) and of BTV8 were successfully used to rescue BTV1 [16], [17] and rgBTV8 [17]. Here, T7-derived RNA transcripts of these two genome segments were used to complete the set of ten RNA transcripts for the second transfection. Transfection supernatants were collected after two days, at which clear CPE was observed for ‘serotyped’ BTVac-1 and BTVac-8. After virus stocks were prepared on BSR cells (one passage), viruses were characterized by S2-genotyping for serotype 1, 6 and 8 and by PCR with primers specific for the respective Seg-6 of BTV1, −6 or −8. The presence of both Seg-2 and Seg-6 were confirmed for the respective BTVac-x viruses (not shown).

### Growth characteristics of BTVac-1, BTVac-6 and BTVac-8 on BSR cells

**Figure 2:**
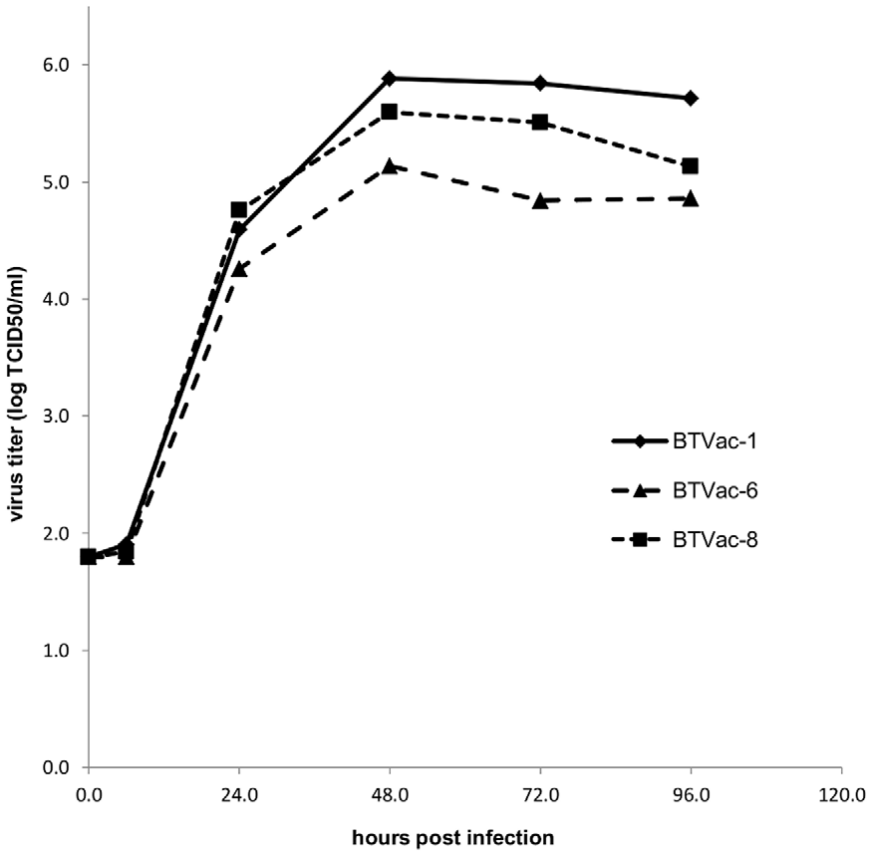
Growth characteristics of BTVac-1, BTVac-6 and BTVac-8 on BSR cells. Virus was infected on confluent monolayers of BSR cells grown in 2 cm^2^ wells with an MOI of 0.1. Total virus titers were determined at 0, 6, 24, 48, 72 and 96 hours post infection and expressed as tissue culture infective dose (log^10^TCID_50_/ml).

The originating BTVac-6 or rgBTV6 and ‘serotyped’ vaccine viruses BTVac-1 and BTVac-8 were compared for growth on BSR cells (Fig. 2). Initial virus titers at 6 hpi showed no increase and was below the detection level of 1.8 log^10^TCID_50_/ml. At 24 hpi, both BTVac-1 and BTVac-8 grew to slightly higher virus titers (4.59 and 4.76 log^10^TCID_50_/ml) compared to 4.26 log^10^TCID_50_/ml for BTVac-6. Maximum virus titers for BTVac-1, BTVac-6 and BTVac-8 were reached after 48 hours (5.88, 5.14 and 5.6 log^10^TCID_50_/ml, respectively). The differences in virus titers as measured at 48 hpi remained for 72 and 96 hpi. Apparently, BTVac-1 and BTVac-8 seemed to grow to slightly higher titers on BSR cells than the originating rgBTV6 (BTVac-6).

### Vaccination/challenge experiment with serotyped BTV viruses

**Figure 3:**
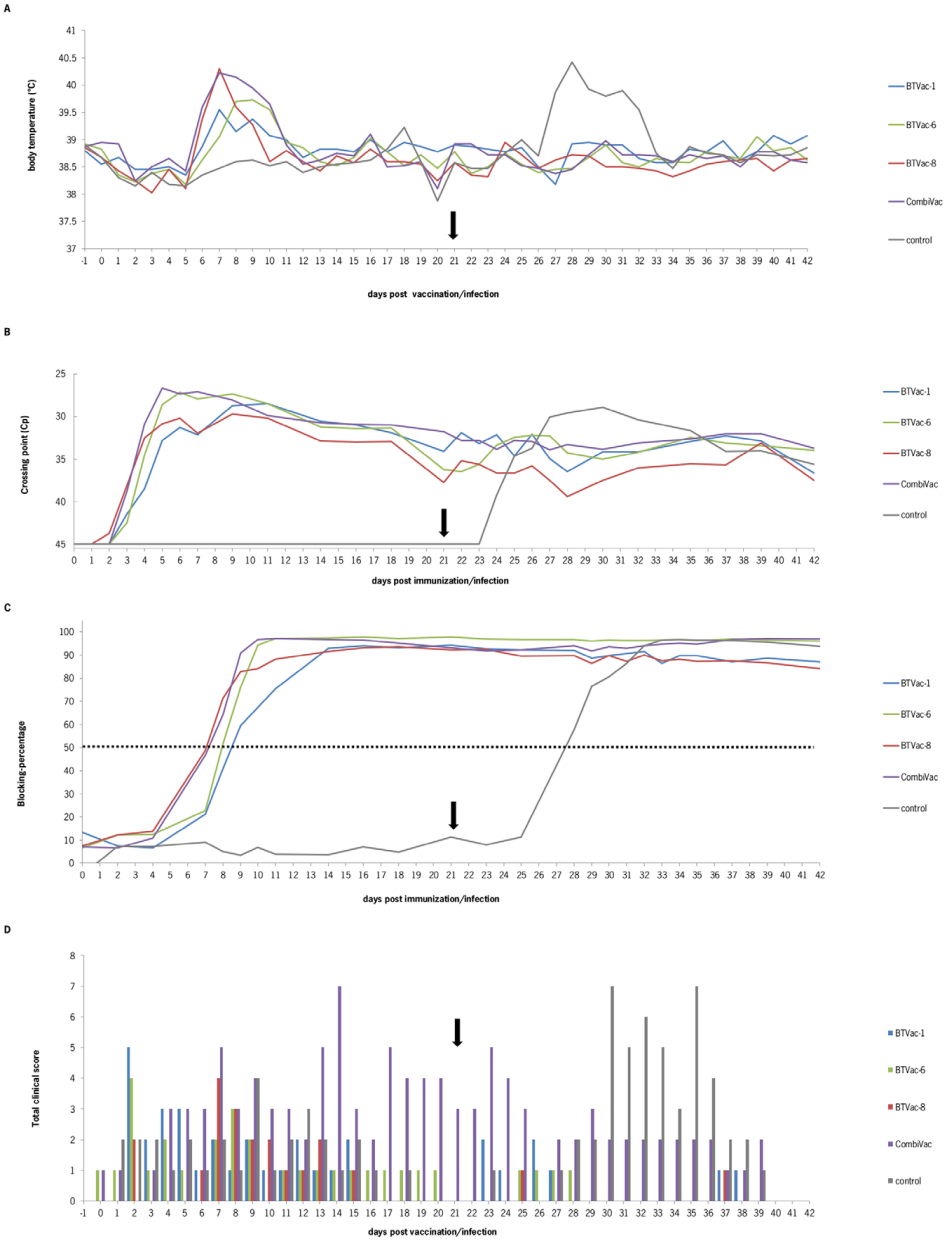
Vaccination/challenge experiment with serotyped BTV viruses. On day 0 (0 dpi), four sheep were intramuscularly (i.m.) vaccinated in the neck with 1 ml of 10^5^ TCID_50_/ml BTVac-1, 1 ml of 10^1.4^ TCID_50_/ml BTVac-6, or 1 ml of 10^5^ TCID_50_/ml BTVac-8. A fourth group was vaccinated i.m. with 1 ml in total consisting of 0.33.10^5^ TCID_50_/ml BTVac-1, 0.33.10^1.4^ TCID_50_/ml BTVac-6 and BTVac-8. On 21 dpv (arrow), all sheep, including the control group were challenged subcutaneously (s.c.) with 1 ml of 10^5^ TCID_50_/ml of BTV8/net07/e1/bhkp3. (A) body temperatures (mean values per group; lines) were recorded daily. (B) BTV was determined by the panBTV-PCR test (mean values per group). (C) BTV-VP7 directed antibodies in serum samples were detected with the panBTV blocking ELISA. The mean blocking percentage per group was displayed as 100-value (sample). (D) Clinical scores were observed daily and displayed as total scores per group (bars per group).

Vaccinated sheep developed fever for one or more days between 6 and 11 dpv (Fig. 3A). The increased body temperature in sheep of the BTVac-6 group was observed approximately one day later than in the other vaccinated groups. Vaccinated sheep were tested positive by the panBTV-PCR test from 3 dpi onwards (Fig. 3B), except for one sheep in the BTVac-1 and in the BTVac-8 group, which became positive for BTV RNA only after 11 dpv. This corresponds with fever readings only on 11 dpv for these particular animals. All animals remained PCR positive, except for one animal in the BTVac-8 group, which is the same animal with the delayed fever and delayed PCR-positivity. All animals seroconverted for BTV VP7 antibodies between 7–9 dpv (Fig. 3C). All vaccinated animals developed very mild clinical signs like nasal/ocular discharge and/or upper airway distress (Fig. 3D). Sheep in the CombiVAc group had the same very mild clinical signs but for a longer period. All sheep of the control group remained negative in PCR and ELISA (Fig. 3B–C). None of the sheep showed fever and only very mild signs were observed. (Fig. 3A and D).

After challenge with virulent BTV8/net07e1/bhkp3 [17], nonvaccinated sheep of the control group developed mild to moderate clinical signs of BT (Fig. 3D), except for one sheep which showed no clinical signs at all. More severe clinical signs were observed from 9 days post challenge (dpc) onwards. Two of the animals were listless, showed local oedema and red mucous in the mouth for several days. All sheep in the control group developed fever between 6 and 11 dpc. All sheep became PCR-positive between day 3–6 dpc and remained PCR-positive until the end of the trial (21 dpc) (Fig. 3B). Sheep seroconverted by ELISA from 7 dpc onwards (Fig. 3C).

Sheep in all vaccinated groups did not develop fever after challenge (Fig. 3A). Vaccinated sheep showed very mild clinical signs and sheep vaccinated with the CombiVAc showed slightly more clinical signs after challenge (Fig. 3D). Since sheep in this group still showed clinical signs at challenge, it is unclear whether this was solely due to the challenge, or an accumulation of clinical signs caused by challenge and vaccination.

On 21 dpv, all sheep in the BTVac-1 group and the BTVac-6 group were tested positive for the respective S2-genotyping, whereas S2-genotyping was negative for serotypes not used for vaccination (Table 2). For the BTVac-8 group, three out of four sheep were tested positive for serotype 8, and all four were negative for both serotype 1 and 6. The fourth sheep was tested positive at 11 dpv for serotype 8, which corresponds to the results of the panBTV-PCR test for this particular animal (data not shown). Three out of four sheep in the CombiVac group were tested positive for serotype 1 and 8, whereas only one sheep was positive for serotype 6 at 21 dpv. At 11 dpv, all sheep were tested positive for both serotype 1 and 8, but not for serotype 6 (data not shown). After challenge, results for vaccinated animals were very similar to results as obtained on 21 dpv/0 dpc Table 2). For serotype 1 and 6, two sheep in the CombiVac group were tested positive by the respective S2-genotyping. For serotype 8, three sheep were positive by S2-genotyping for serotype 8 in both the CombiVac group and the BTVac-8 group. However, since Seg-2 is the same for BTVac-8 and BTV8/net07, there is no discrimination by S2-genotyping between vaccine virus and challenge virus in these groups. Thus, no conclusion can be drawn about viremia of BTV8/net07 in the BTVac-8 group and the CombiVac group.

**Table 2:** Genotyping results from vaccinated and challenged animals with serotype specific PCR tests on Seg-2 (S2-genotyping) and restriction enzyme analysis of amplicons of the panBTV-PCR test (S10-genotyping).

Group (n = 4)		S2-genotyping (number of positive animals)^a^									S10–genotyping^b^					
		11 dpv			21 dpv			14 dpc			7 dpc		11 dpc		14 dpc	
	Serotype	1	6	8	1	6	8	1	6	8	BT Vac-x	BTV8	BT Vac-x	BTV8	BT Vac-x	BTV8
BTVac-1	1	4	0	0	4	0	0	4	0	0	+		+		+	
BTVac-6	6	0	4	0	0	4	0	0	4	0	+		+		+	
BTVac-8	8	0	0	4	0	0	3	0	0	3	+		+		+	
CombiVac	1, 6, 8	4	0	4	3	1	3	2	2	3	+		+		+	
Control	8	0	0	0	0	0	0	0	0	4		+		+		+

a. S2-genotyping was based on a serotype specific PCR-test with specific primer sets listed in table 1 [14].

b. S10-genotyping was based on the panBTV-PCR test used for diagnostic purposes [23]. The amplicon was digested with specific restriction enzymes and analyzed on an agarose gel, see materials and methods.

Sheep were not tested by S10-genotyping prior to challenge with BTV8/net07. After challenge, samples of vaccinated sheep on 7, 11 and 14 dpc were tested positive for S10-genotyping for BTVac-x viruses, whereas all were negative for BTV8/net07 (Table 2). These results confirmed the findings by S2-genotyping and indicates that challenge virus BTV8/net07 was not replicating in all vaccinated groups. As expected, S10-genotyping of samples from the control group of all three tested days after challenge were positive for BTV8/net07 and negative for Seg-10 of BTVac-x viruses.

The sensitivity of this test was determined by a mixing experiment of defined amplicon material from BTV6 and 8 (Fig. 1). It was shown that at least 16 ng of digested amplicon could be detected. The detection limit for undigested material was approximately 5 ng of amplicon DNA (data not shown) and corresponds with a Cp signal of 31–33 in the panBTV-PCR test. In conclusion, complete digestion of amplicon with the BTV6-specific restriction enzyme confirmed the absence of undigested amplicon, and consequently the absence of challenge virus BTV8/net07.

Serotype specific neutralizing antibody (nAb) titer in the different groups of sheep were determined by SNT with different BTV serotypes (Table 3). Sheep vaccinated with BTVac-1 developed nAb titers (1∶80 to 1∶3,840) against BTV1 on 21 dpv, whereas no nAb titers (<10 to 15) were found for BTV6 and very low nAb titers (15 to 20) against BTV8. One sheep in this group (4908) developed a significantly higher nAb titer (480) against BTV8. On 42 dpv/21 dpc, nAb titers against BTV1 were between 1∶10 and 1∶960, whereas titers for BTV6 were very low (1∶10 to 1∶30). No significant increase of nAb titers against BTV8 was found in individual sheep after challenge, although a slightly increase of nAb titers against BTV8 could be suggested. All sheep in the BTVac-6 group developed significant nAb titers (1∶160 to 1∶640) on 21 dpv/0 dpc against BTV6, whereas very low nAb titers (1∶10 to 1∶20) were measured for BTV1. nAb titers against BTV8 varied between 1∶40 and 1∶320. On 42 dpv/21 dpc, low nAb titers against serotypes 6 and 8 were measured (1∶40 to 1∶160), and no nAb titers (<10) were detected against BTV1. All sheep vaccinated with BTVac-8 group developed significant nAb titers (1∶480 to 1∶2,560) against serotype 8 on 21 dpv, whereas no nAb titers (<10 to 1∶15) against serotypes 1 and 6 could be measured. On 42 dpv/21 dpc, nAb titers were similar as before challenge and varied between 1∶80 to 1∶2,560 for serotype 8, and not detectable to very low (<20) for serotypes 1 and 6. In the CombiVac group, nAb titers were measured on 21 dpv against BTV1 (1∶20 to 1∶1,280) and BTV8 (1∶160 to 1∶5,120), but were not detectable (<10 to 1∶15) for BTV6. On 42 dpv/21 dpc, nAb titers against BTV1 (1∶320 to 1∶1,280) and BTV8 (1∶160 to 1∶1,280) were measured. For serotype 6, two out of four animals had developed a nAbs titer against BTV6 (sheep 4913 and 4915). These sheep were also positive by S2-genotyping for serotype 6 (data not shown). Sheep in the control group had no nAbs titers prior to challenge with BTV8/net07. On 21 dpc, nAb titers for BTV8 ranged from 1∶960 to 1∶3,840, whereas no nAb titer was measured against serotypes 1, and 6, except for sheep 4917 (nAb titer of 1∶160 against BTV1).

**Table 3:** nAb titres by serum neutralizing tests (SNTs), clinical reaction indexes (CRIs) and the percentage of protection.

Group	Animal number	SNT titres (day) against BTV1, BTV6 or BTV8^a^						CRI^b^	% protection^c^
		21 (0 dpc)			42 (21 dpc)				
		BTV1	BTV6	BTV8	BTV1	BTV6	BTV8		
BTVac-1	4905	80	15	15	10	20	20	0	100
	4906	120	<10	15	30	10	20	0	100
	4907	640	15	20	960	20	80	1	87.5
	4908	3840	10	480	160	30	640	1	87.5
	Mean ± SE							0.5±0.29	93.8±3.6
BTVac-6^d^	4901	<10	640	80	<10	160	40	0	100
	4902	10	640	320	<10	80	120	1	87.5
	4903	20	160	<10	<10	160	40	0	100
	4904	10	320	40	<10	40	40	1	87.5
	Mean ± SE							0.5±0.29	93.8±3.6
BTVac-8	4909	<10	<10	480	<10	20	80	3	62.5
	4910	<10	<10	480	<10	10	480	2	75
	4911	<10	<10	640	15	20	320	0	100
	4912	<10	10	2560	<10	30	2560	0	100
	Mean ± SE							1.25±0.75	84.4±9.4
CombiVac^e^	4913	1280	15	160	1280	640	480	2	75
	4914	20	<10	960	320	10	1280	3	62.5
	4915	20	15	640	480	320	160	2	75
	4916	30	<10	5120	480	<10	1280	0	100
	Mean ± SE							1.75±0.63	78.1±7.9
control	4917	<10	<10	<10	<10	15	1280	6	NA
	4918	10	10	15	160	10	3840	9	NA
	4919	<10	<10	<10	<10	<10	2560	5	NA
	4920	<10	<10	<10	<10	<10	960	12	NA
	Mean ± SE							8.0±1.58	

a. The respective serum neutralization titres were determined against 30–300 TCID_50_ of the indicated BTVs and expressed as the dilution of serum giving a 50% neutralisation endpoint.

b. The clinical reaction index (CRI) was determined according to Huismans et al [24]. See also materials and methods.

c. The percentage of protection was determined as 100-RR, in which RR is the CRI of each sheep expressed as a percentage of that of the average control.

d. BTVac-6 was previously named rgBTV6 [17].

e. CombiVac is a combination of BTVac-1, BTVac-6, BTVac-8.

After challenge, clinical scores of individual sheep were calculated as clinical score index (CRI) (Table 3). Sheep of the control group had a mean CRI of 8±1.58. Sheep in all vaccinated groups showed a strong reduction of the CRI, although some mild clinical signs were observed. The sheep in all vaccinated groups showed a strong reduction in CRI although some mild clinical signs were observed. Means CRI's varied from 0.5±0.29 for BTVac-1 and BTVac-6, 1.25±0.75 for BTVac-8 and 1.75±0.63 for the CombiVac group. Mean protection indices varied from 78% (CombiVac), and 84% (BTVac-8) to 94% for both BTVac-1 and BTVac-6.

Summarizing, BTVac-x vaccine viruses are protective in sheep on three weeks after a single vaccination. Vaccination results in a very strong reduction of clinical disease and completely blocks viremia of virulent BTV8/net07 in sheep.

## Conclusions and Discussion

The incursion of bluetongue virus serotype 8 (BTV8) in North-Western Europe was firstly detected in the Netherlands in August 2006, and has resulted in one of the largest recorded BT outbreaks. The threat of (re)emergence of a BTV serotype needs a quick response to supply effective vaccines. There is a long record of development and application of inactivated and live-attenuated or modified-live BT vaccines, which both have advantages and disadvantages [26], [27], [28]. Further, experimental vaccines have been developed by different approaches, reviewed in Noad and Roy [29]. Recently, reverse genetics was developed for BTV1 [16], BTV1 and BTV8 [7] and BTV1, BTV6 and BTV8 [17] which can be used to further explore the knowledge of BTV and improve current BT vaccines.

Vaccine-related BTV6/net08 has appeared to be avirulent in the field and by experimental infection of different ruminant species [30], [31]. Further, BTV6 regenerated by reverse genetics rgBTV6 (in this study named BTVac-6) was indeed avirulent [17]. Therefore, we selected this avirulent BTV strain as genetic backbone for vaccine development and changed the serotype by exchange of Seg-2 and Seg-6 for these of BTV1 and BTV8. Seg-2 and Seg-6 encode for the outer shell proteins VP2 and VP5. In particular VP2 induces a protective humoral neutralising immune response, which is highly specific for the respective serotype (see references in: [29]). By this method, BT vaccine for another serotype can be made rapidly by exchange of two segments from circulating or (re)emerging BTV serotypes.

The two outer capsid proteins VP2 and VP5 are responsible for virus entry into the host cells. In mammalian cells BTV entry proceeds via virus attachment to the cell, followed by endocytosis and release of a transcriptionally active core particle into the cytoplasm [32], [33], [34], [35], [36]. The structural features of VP2 (propeller-like spike) and VP5 (globular) of the outer capsid correlate with their biological roles in virus entry into the cells [6]. The most exposed BTV protein VP2 is the highly variable protein among BTV serotypes and is the determinant of the serotype. Antibodies raised against VP2 neutralise virus infectivity supporting the fact that VP2 is the cellular receptor binding protein of the virus [37]. The globular outer capsid protein VP5 is likely to be the membrane penetration protein. VP5 protein shares certain secondary structural features with the fusion proteins of enveloped viruses, indicating that it may play a role in virus penetration activity [38]. The outer shell proteins VP2 and VP5 of BTV6 were exchanged with that of BTV1 or BTV8 and therefore this might influence some of the functions mentioned above like infectivity and/or virulence.

In this study two ‘serotyped’ BTVs were generated by completing the backbone of BTV6 (eight segments) with Seg-2 and Seg-6 from BTV1 or BTV8 resulting in BTVac-1 and BTVac-8, respectively. In line with this, rgBTV6 [17] was re-named BTVac-6 in this study. Rescued vaccine viruses (BTVac-x; x presents the serotype) were characterized and compared.

Still, initial virus titres were comparable (within 0.5 log^10^ TCID_50_/ml) at 24 hpi for the generated BTVac-x viruses. The maximum titre of BTVac-1 was even slightly higher (0.2–1 log^10^ TCID_50_/ml) at all following time points (48, 72 and 96 hpi), whereas the original BTVac-6 was the lowest at each of the following time point (Fig. 2). Apparently, these outer shell proteins fit well on the BTV6 core particle. Since the difference between these BTVac-x viruses is limited to Seg-2 and Seg-6, it can be concluded that the observed small growth advantage for the two serotyped viruses is caused by VP2 and/or VP5. It is unknown whether this small difference in virus replication *in vitro* also reflects a difference in virus replication *in vivo*.

Unfortunately, a non-intended lower dose of BTVac-6 was used for vaccination of sheep compared with respect to the ‘serotyped’ vaccine viruses BTVac-1 and BTVac-8. Although the sheep of the BTVac-6 group were slightly delayed in developing fever and PCR-positivity, all sheep seroconverted by ELISA between 7–9 dpi (Fig. 3C) which was comparable to the other vaccinated groups. This indicated that the lower dose of 1 ml of 10^1.4^ TCID_50_/ml BTVac-6 is sufficient to vaccinate animals and resulted in comparable seroconversion with respect to vaccination with 1 ml of 10^5^ TCID_50_/ml of BTVac-1 or BTVac-8. As a consequence of the lower titre of the virus stock, the one-third amount of BTVac-6 in the CombiVac group was also lower and was much less than for the other two BTVac-x vaccine viruses. Apparently, the amount of BTVac-6 in this combination was limited, since BTVac-6 could not be detected in all vaccinated sheep (Table 2). Further, two out of four sheep did not raise significant nAb titres specific for serotype 6, and the two other sheep showed only very low nAb titres for serotype 6 (Table 3). From these data, however, it is not clear whether this is caused by the limited amount of BTVac-6, negative interference by the excess of other BTVac-x viruses, or by the slightly slower replication rate of BTVac-6 as observed *in vitro*.

Besides fever, only very mild clinical signs (Fig. 3D) were observed in vaccinated sheep, which was also seen in experimental infections of BTV6/net08 and rgBTV6 (here named BTVac-6) [14], [17]. It can therefore be concluded that VP2 and VP5, of which these originated from virulent BTV8/net07 in BTVac-8, are not associated with virulence. Sheep in the CombiVac group showed similar clinical signs but for a longer period of time. Likely, sheep react stronger after vaccination with immunogenic different viruses and/or needs more time to recover from vaccination. For future research, this observation should have to be taken into account in the light of combining BTVac-x vaccine viruses.

Sheep of the control group showed several days with fever after challenge with BTV8/net07, whereas vaccinated sheep did not. The control group had an average CRI of 8, whereas average CRI's of the vaccinated groups were <2. This indicated that one single vaccination clearly reduce clinical disease in sheep. Sheep in the CombiVac group seemed to be a little less protected (Table 3), but it is unclear whether the measured CRI's after challenge are caused by challenge virus, and thus are less protected by CombiVac, or are the result of prolonged clinical reaction by CombiVac vaccination, or are the sum of both.

No challenge virus was detected in vaccinated sheep, indicating that a single vaccination completely blocks replication of challenge virus. No seroconversion or booster of the humoral neutralising immune response specific for serotype 8 was measured for vaccinated sheep after challenge. This confirms the absence of replication of the virulent challenge virus BTV8/net07.

Prior to challenge, vaccinated animals developed significant nAb titres against the respective serotype and very low nAb titres or none at all for the other serotypes. Still all vaccinated sheep, irrespective of the used vaccine virus, were protected from challenge with BTV8/net07, thus including the groups vaccinated with BTVac-1 and BTVac-6. We suggest that cross-protection as observed in this study is due to a nonspecific cell-mediated immune response. Cross-protective immune response to BTV has been described [39]. This cross-protective response involves VP2 and NS1 as major ovine CTL immunogens of which NS1 is cross-protective and VP2-specific CTL responses are not [40].

A longer time period, e.g. four weeks or more, after vaccination with live-attenuated vaccine will show serotype specific protection. Anti-BTV CTL's showing serotype cross-reactivity have been demonstrated to peak between 7 and 21 days after infection [40], [41], [42] and even extends to 66 days after multiple immunization [40]. Still, the detected serotype specific nAb titre after vaccination with BTVac-x viruses is very promising in the light of long lasting protection for the respective serotypes, and can be expected to be very similar as for live-attenuated vaccines.

The selected vaccine virus background BTVac-x is safe, by which x represents the serotype of the outer shell proteins, even if completed with outer shell proteins of virulent BTV8/net07 (Fig. 3). Apparently, no virulence markers are located on VP2 or VP5 of BTV8/net07. Small differences in nAbs titres could be caused by the lower dose (10^1.4^ TCID_50_/ml) for BTVac-6 than for the other vaccinated groups (10^5^ TCID_50_/ml). Further, for combination vaccines consisting of BTVac-x viruses, the amount of each BTVac-x have to be comparable, since negative interference between different BTVAc-x viruses could be expected resulting in a less pronounced viremia and a lower level of nAbs titres (Tables 2 and 3).

The quick generation of BTVac-x vaccine viruses by use of the genetic backbone of vaccine-related BTV6/net08 can also be used to generate safe BTVac-x vaccine virus for other serotypes. Serotyping of rgBTV6 (BTVac-6) presented here for serotypes 1 and 8 is promising for serotyping for other BTV serotypes. Indeed, ‘serotyping’ can be extended for other serotypes but is not unlimited. We were able to ‘serotype’ for TOV, the proposed 25^th^ serotype [3], and a few others but not for all BTV serotypes [43]. In conclusion, these results show the strategy to develop faster BT vaccines for desired BTV serotypes. However, issues regarding compatibility of Seg-2 and Seg-6 from other serotypes need to be addressed, in particular with respect to protein-protein interactions between these outer shell proteins and the core of BTV6/net08.

Since both live-attenuated and inactivated vaccines currently have a history of safety issues, improvement of safety is an important issue. Recently, protection against BTV8 by replication-defective BTV1 with VP2 of BTV8 was shown [44]. These data indicate that VP2 of the respective serotype is sufficient to induce protection, although nonhomologous challenge was not included in this study. Thus, serotype-independent protection could be involved as observed for BTVac-x vaccine viruses in this study. BTVac-x vaccine viruses can be easily generated and are effective in the induction of serotype-specific nAbs as soon as 21 dpi after a single vaccination. Most likely, these tested BTVac-x vaccine viruses will be protective for their respective serotype, like it has been observed for traditionally generated live-attenuated BT vaccines.

Development of DIVA vaccines (DIVA: Differentiating Infected from Vaccinated Animals) is of significant importance to control Bluetongue. BTVac-x vaccines induce a complete immune response against the respective serotypes. Consequently, animals vaccinated with BTVac-x vaccines cannot be distinguished by serological testing from animals infected by the respective BTV serotype. In 2008, BTV1 and BTV6 were detected by genotyping on Seg-10 in the infected and vaccinated ruminant population for BTV8 [14]. This method is irrespective of the serotype, and combines the high-throughput panBTV PCR assay [23] and sequencing of amplicons between the PCR-primer positions. Here, we have improved this method for routine use to differentiate between virulent BTV8 and BTVac-x vaccines. Note that all BTVac-x vaccines have the same genetic background of BTV6, and that field BT-viruses will be detected/identified by the high genetic variation in Seg-10. Thus, BTVac-x vaccines in combination with genotyping on Seg-10 are DIVA-vaccines to detect infectious animals in vaccinated populations.

The system of tailor-made vaccines by exchange of outer shell proteins for those of other serotypes in a defined avirulent genetic backbone offers more advantages, like fully defined genomes, and similar growth characteristics *in vivo* and *in vitro*. Moreover, these ‘serotyped’ BTVac-x vaccine viruses share eight out of ten genome segments, and consequently the chance on new undesired (virulent) reassortants after mixing these, e.g. for multi-serotype vaccines, is negligible. In addition, the proteins VP2 and VP5 that differ between BTVac-x vaccine viruses do not harbor virulence markers for the tested BTV serotypes. Further, due to the same replication machinery, negative interference between BTVac-x vaccine viruses after vaccination is reduced to a minimum, although equal amounts of each BTVac-x virus seems to be important as shown in this study. Finally, by the similar growth characteristics *in vitro* of BTVac-x viruses, similar costs for virus production of each BTVac-x virus could be expected. Noteworthy, these BTVac-x viruses can be used as live-attenuated BT vaccine or, in order to address safety issues, as inactivated BT vaccine. Research is in progress to start ‘serotyping’ of BTV6/net08 for other serotypes in order to develop virus stocks of more BTVac-x viruses for vaccine production.

## Supporting Information

Table S1 Clinical score table BTV animal trial. The clinical signs described in this table were based on findings in the field [45] as well as experimental data [22] and scored daily depending on severity from 0–3 points during 3–15 days post inoculation.
(DOCX)
